# Conservative Management of Dentigerous Cysts Associated With Mixed Dentition: A Retrospective Cohort Study

**DOI:** 10.7759/cureus.47143

**Published:** 2023-10-16

**Authors:** Soha Alqadi, Safa A Jambi, Abdel Aziz B Abdullah, Fadia M Aljuhani, Shadia A Elsayed

**Affiliations:** 1 Pediatric Dentistry and Orthodontics, College of Dentistry, Taibah University, Madinah, SAU; 2 Pediatric Dentistry and Orthodontics, Taibah University, Madinah, SAU; 3 Oral and Maxillofacial Surgery, Al-Azhar University, Assuit, EGY; 4 Oral and Maxillofacial Surgery, Taibah University, Madinah, SAU; 5 Oral and Maxillofacial Surgery, Al-Azhar University, Cairo, EGY

**Keywords:** odontogenic, pediatric, mandible, cyst, marsupialization

## Abstract

Objectives

The pediatric dentigerous cysts might vary by region and population group. Large cystic lesions are typically treated with marsupialization before enucleation in order to decompress the lesion and reduce its volume; however, in pediatric cystic lesions, conservative marsupialization and decompression can be used to manage the condition without additional enucleation. The current study's objectives were to present a case series of pediatric dentigerous cysts and discuss the conservative management of these cystic lesions associated with mixed dentition.

Methods

A retrospective cohort analysis of patients diagnosed with cystic lesions between 2016 and 2023 was identified. Data on clinical, radiological, pathological, and odontogenic causes were collected. The marsupialization approach was performed in all cases. Patient demographic information was also examined, and a literature review was carried out to identify comparable cases.

Results

Sixteen young patients were diagnosed with dentigerous cysts, and this was confirmed by clinical, radiological, and pathological examinations. Females comprised 56.2% of the cases, with the right side predominating (62.5%). Deciduous teeth related to the lesion could be extracted normally in all cases. All associated permanent teeth erupted rapidly after the intervention.

Conclusion

The marsupialization technique used in the current cases of dentigerous cysts associated with mixed dentition was highly successful, and all permanent impacted teeth erupted without any cystic recurrence.

## Introduction

A dentigerous cyst (DC), also known as a follicular cyst, is an odontogenic cystic type with an epithelial lining connected to the cementoenamel junction associated with an unerupted tooth, preventing it from erupting and causing displacement to the inferior border [1]. It is the second-most frequent odontogenic cyst after radicular cyst and the most frequent developing cyst [2,3]. In Saudi Arabia, the prevalence of DC is 25.11%, and it primarily affects men, with the greatest incidence occurring between the second and third decades of life [4]. The majority of DCs are thought to be developmental in nature, resulting from fluid collection between the reduced enamel epithelium and the enamel or between the layers of the enamel organ [5,6]. Several studies have found DC of permanent teeth in conjunction with infected deciduous teeth [7,8]. Bilateral lesions are typically associated with syndromes such as basal cell nevus syndrome, mucopolysaccharidosis, and cleidocranial dysplasia [9-11].

The treatment of DC is determined by the patient's age, location, and extent of the lesion [4,12]. The affected teeth are frequently extracted and enucleated as part of the treatment. In the case of large cystic lesions, marsupialization is performed first, followed by enucleation [13]. Conservative treatment with removal of deciduous teeth followed by marsupialization to allow spontaneous eruption of permanent teeth has a better prognosis in mixed dentition [5,14,15].

The pattern of pediatric dentigerous cysts might vary by region and population group. It is important to emphasize a differential diagnosis with other similar patterns, including keratocysts, unicystic ameloblastomas, ameloblastic fibromas, central giant cell granulomas, and large radicular cysts. Large cystic lesions are typically treated with marsupialization before enucleation in order to decompress the lesion and reduce its volume; however, in pediatric cystic lesions, conservative marsupialization and decompression can be used to manage the condition without additional enucleation. Therefore the current study aimed to present a case series of pediatric dentigerous cysts and discuss the conservative management of these cystic lesions associated with mixed dentition.

## Materials and methods

Study design

The current retrospective cohort study was carried out at oral surgery dental clinics, where patients with cystic lesions related to unerupted permanent mandibular teeth associated with retained deciduous teeth were referred from the pedodontic department to the oral and maxillofacial department for surgical management between December 2016 and June 2023. The study's protocol was approved by the Al-Azhar Institutional Ethics Committee, and it was carried out in accordance with the Helsinki Declaration for Research Studies (AUAREC202300009-15).

Selection criteria and data collection

Patients younger than the age of 14 who received conservative management for dentigerous cysts associated with deciduous dentition and had completed their follow-up were included. Patients whose demographic or surgical data were missing or incomplete had been excluded from the study.

**Figure 1:**
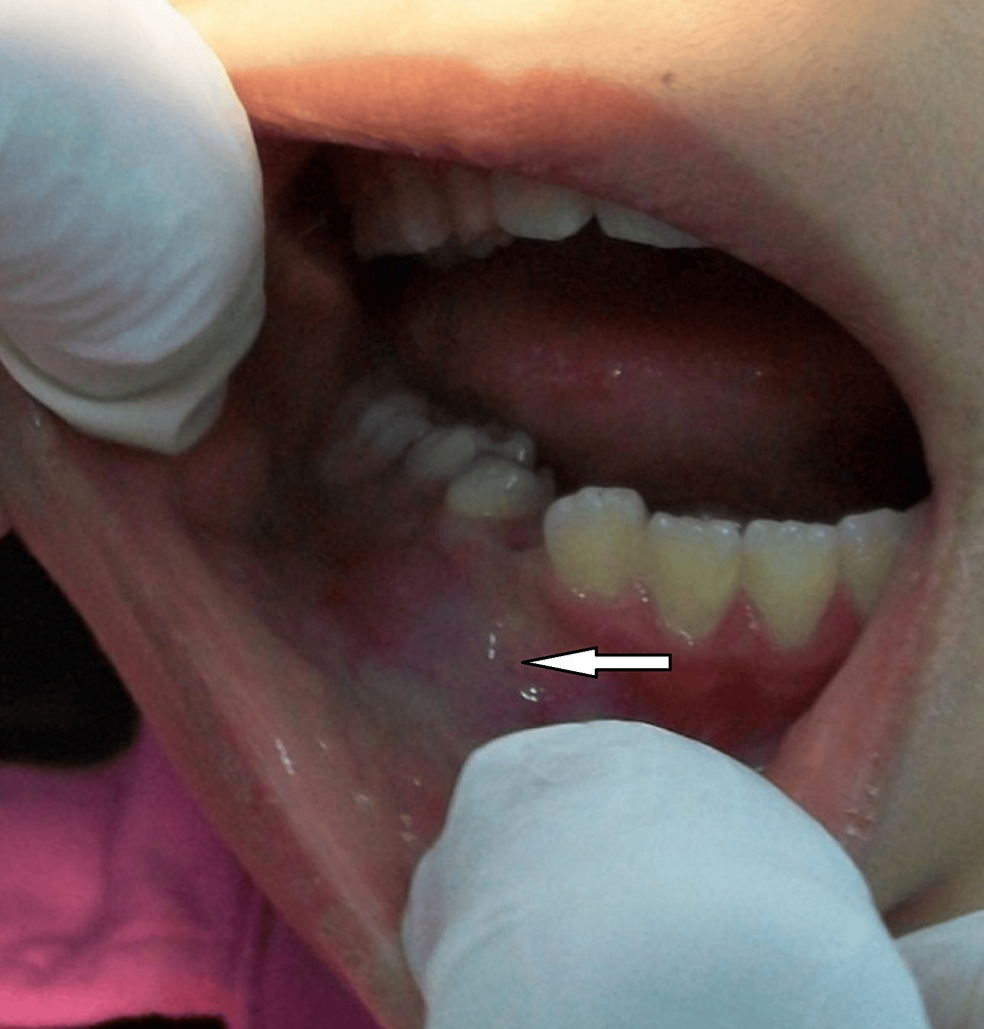
Intraoral photograph showing swelling at the buccal vestibule of the right premolar region

Thorough clinical recorded investigations in all included cases indicated intraoral swelling, bulging buccal and superior cortex, and normal colored mucosa. On palpation, all patients reported mild pain (Figure 1).

A radiographic examination based on an extraoral orthopantomogram revealed a well-defined radiolucency surrounding the coronal region of the permanent unerupted teeth and connected to the root apices of deciduous teeth.

Surgical approach

Informed consent was obtained. Before surgery, all diagnosed cases had an aspiration test that revealed clear yellowish cystic fluid. The surgical procedure was carried out under local anesthesia. Blocking the mandibular nerve and using infiltration anesthesia for hemostasis.

**Figure 2:**
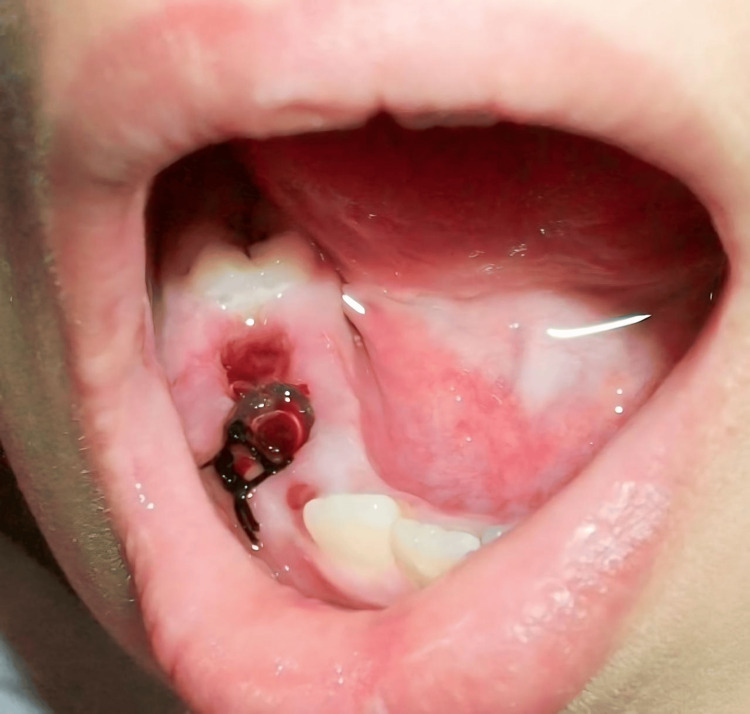
Intraoral photograph showing fixation of the rubber tube after extraction of the retained deciduous teeth

After the associated deciduous tooth extraction, the socket was used to obtain access to the cystic lesion. A good opening into the cystic cavity is created by removing part of the cystic wall. We trimmed a polyethylene tube to fit the size of the cystic opening using catheters or tubing that are commonly present in hospitals, we kept the opening open by suturing the tube to the adjacent gingiva all through around with 3-0 silk suture material. Through this tube opening, we had easy access to daily irrigation and continual draining of the cystic lesion with normal saline (Figure 2).

To monitor the healing process, Patients were instructed to continue irrigation at home five times per day after the first week of daily visits to the outpatient surgical clinic to irrigate the cystic cavity with normal saline through the tube opening.

The tube was quickly extruded, and the rubber tube was removed after 15 days. The eruption of the permanent impacted teeth was accelerated and permanent teeth erupted in all patients. When the cystic lesion was associated with more than one unerupted tooth (12.5% of the cases), additional orthodontic treatment was necessary.

Statistical analysis

Data were collected from clinical patient records that had been saved and processed in a separate Excel spreadsheet (Microsoft Corporation, Redmond, WA). Mean and standard deviation for continuous values and frequencies and percentages for categorical variables were used to summarize descriptive statistics. To compare the effect of time on two categorical variables (gender and side), chi-square tests were used.

## Results

The study included 16 patients ranging in age from 8 to 13 years old, with a mean age of 10.12±1.5. Retained deciduous teeth were often found in the premolar region. Seven (43.8%) were found in the second mandibular premolar area followed by the first mandibular premolar area.

**Figure 3:**
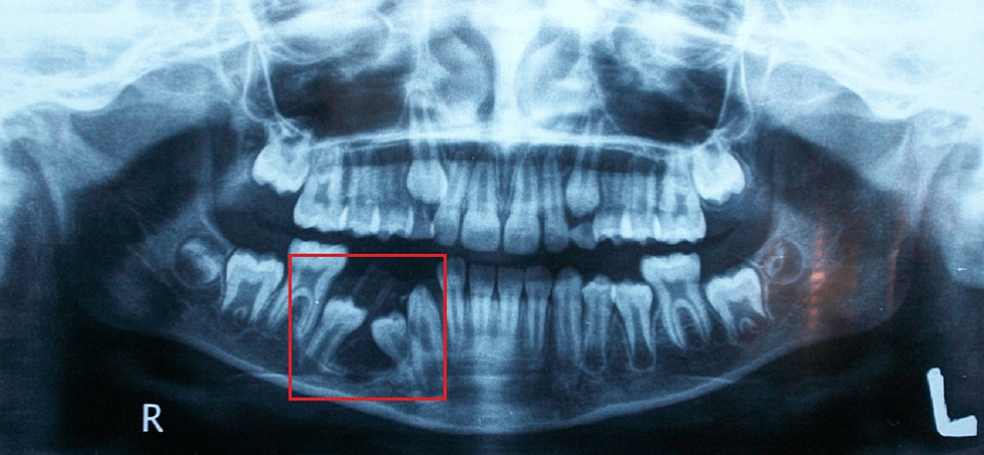
Extraoral orthopantomogram photograph demonstrating the cystic lesion shrinking in size and the impacted permanent teeth beginning to come out and moving away from the inferior border

Females comprised nine of the study cases (56.2%). Ten cysts were found on the right side, which was the most common (62.5%). Radiographically, in the majority of cases, the roots of the associated deciduous teeth showed signs of resorption and cystic lesions, which caused displacement of adjacent structures and neighboring teeth (Figure 3). To relieve pain, analgesic and anti-inflammatory medication (ibuprofen 200 mg for five days) was prescribed, and the patient was instructed to keep mouth irrigation and brush teeth to maintain good dental hygiene during the healing process (Table 1).

**Table 1:** Descriptive characteristics of the study sample

Study variables	Variable category	Frequency	%
Age	Age (Mean/SD)	10.12	1.54
side	Right	10	62.5%
	Left	6	37.5%
	Total	16	100.0%
Related tooth	Canine	1	6.2%
	First premolar	5	31.2%
	Second premolar	7	43.8%
	First molar	1	6.2%
	First and second premolar	2	12.5%
	Total	16	100.0%
Gender	Male	7	43.8%
	Female	9	56.2%
	Total	16	100.0%
Time of eruption (days)	15-20	9	56.2%
	21-25	4	25%
	≥26	3	18.8%

**Figure 4:**
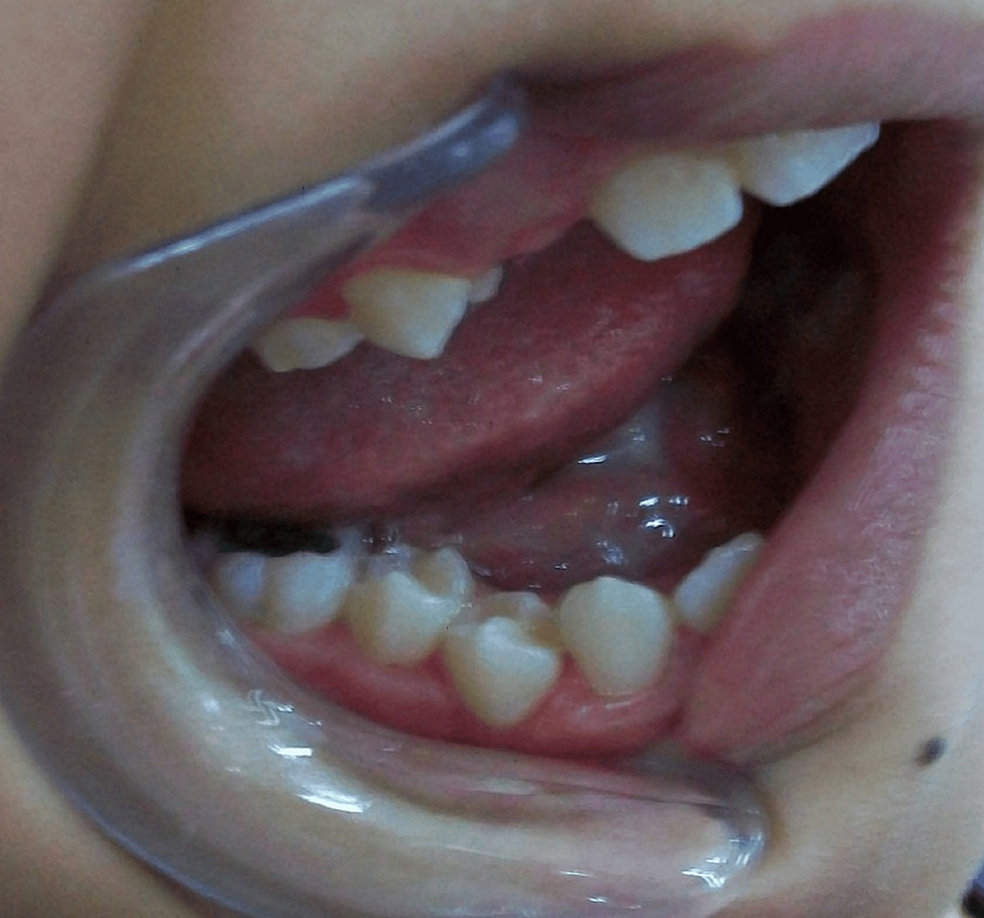
An intraoral photograph demonstrating the permanent teeth's full eruption

All of the patients' cystic lesions healed without complication or infection, and the conservative approach was successful in managing all included 16 cases without recurrence (Figure 4). The chi-square test found that there was no statistically significant association between patient age or gender and the timing of eruption or the tooth side affected (P≥ 0.05).

## Discussion

The current conservative technique aimed to eliminate the cystic lesion while preserving the associated permanent tooth and surrounding structures. It may not be appropriate for all patients, and the treatment approach was determined by a number of factors, including the size of the cyst, the position of the impacted tooth, and the patient's overall medical and dental health [15,16].

In the current cases, a thorough clinical and radiographic evaluation was performed prior to surgical intervention to determine the location, size, and extent of the lesion. It is critical for an oral surgeon to thoroughly analyze the condition and offer the best treatment approach for each individual case.

Large cystic lesions are typically treated with marsupialization before enucleation in order to decompress the lesion and reduce its volume; however, in juvenile cystic lesions, conservative marsupialization and decompression can be used to manage the condition without additional enucleation.

Conservative marsupialization was performed through the extraction site opening only with the aid of this opening through tube suturing in order to avoid the risk of weakening the mandible, as associated permanent teeth were located deeply near the inferior border of the mandible.

DC pathogenesis is uncertain [17]; however, "Main's theory" claims that follicle separation from enamel is caused by venous outflow obstruction caused by the pressure of the erupting tooth on the affected follicle, resulting in fast serum transfer through the capillary walls [18]. Dentigerous cysts can be inflammatory in nature, with inflammation from a non-vital deciduous tooth or other source extending to include the developing tooth's dental follicle and leading to dentigerous cysts [8,13,19].

The mandibular third molars are the most usually affected, followed by the maxillary canines, mandibular second premolars, and maxillary third molars [12,20]. Unless infected, it is asymptomatic and is detected accidentally on radiographs. It has the potential to expand to a great size, resulting in bone enlargement and facial asymmetry [14,21].

A well-defined sclerotic border and a unilocular radiolucent area larger than 5 mm which is connected with the crown of an unerupted tooth on radiographs are the main characteristic diagnostic parameters [22,23]. However, the margins of the infected cysts could be ill-defined [9]. On radiographs of DC, the crown-cyst relationship may typically be seen in three different ways. The most common type is the central variety, in which the cyst encircles the tooth's crown and the crown seems to protrude into the cyst [24]. The lateral variety is most commonly seen in mesioangularly impacted mandibular third molars. The cyst extends along the root surface of the unerupted tooth in the circumferential variant [23].

On radiographs, other odontogenic cysts, such as keratocystic odontogenic tumor, mural ameloblastoma, and adenomatoid odontogenic tumor, may look similar. Compared to other jaw cysts, DC has a higher potential for the root resorption of neighboring teeth [6,16,25]. Histologically, the non-keratinized stratified squamous epithelium that lines the cyst cavity may exhibit various differences. Numerous mucous cells, as well as inflammatory or dysplastic alterations, may be present [3-5].

Although DC recurrence is uncommon, it is possible for it to lead to major side effects such as ameloblastoma, mucoepidermoid carcinoma, and squamous cell carcinoma [6,10,24].

A 10-year-old Saudi girl with DC associated with the right mandibular premolar was described in the Alnofaie 2019 case report. Following their marsupialization procedure, the lesion completely disappeared and the permanent premolar spontaneously erupted during a 13-month follow-up [2].

The presented conservative marsupialization approach was convenient for patients and parents, avoiding the risks of complete enucleation under general anesthesia, preserving the weakened inferior border, and lowering the recurrence rate, and this was achieved gradually through continuous wound care. Furthermore, the approach demonstrated a lower complication rate and facilitated recovery in conjunction with excellent pediatric tissue regeneration [26].

Limitations of the study

The current study provided good observational outcomes, but it had some limitations, including a small number of patients included and the retrospective design of the study, which could still be informative, especially when prospective studies or randomized controlled trials are not feasible.

## Conclusions

We could conclude that pediatric dentigerous cystic lesions associated with mixed dentition are not uncommon among pediatric dental patients. The current approach is recommended for pediatric dentigerous cysts where preserving the associated tooth or adjacent structures is desired and reducing the need for complex surgical procedures or bone grafts.
